# Metabolic crosstalk between roots and rhizosphere drives alfalfa decline under continuous cropping

**DOI:** 10.3389/fpls.2024.1496691

**Published:** 2024-12-12

**Authors:** Yuanyuan Ma, Xiaoping Zhou, Yan Shen, Hongbin Ma, Quanhong Xue

**Affiliations:** ^1^ College of Forestry and Prataculture, Ningxia University, Yinchuan, Ningxia, China; ^2^ Ningxia Rural Science and Technology Development Center, Yinchuan, Ningxia, China; ^3^ College of Natural Resources and Environment, Northwest A&F University, Yangling, Shaanxi, China

**Keywords:** *Medicago sativa* L., biological decline, rhizosphere-enriched metabolites, age-related metabolites, metabolic pathway

## Abstract

Considerable biological decline of continuously cropped alfalfa may be tightly linked to rhizosphere metabolism. However, plant-soil feedbacks and age-related metabolic changes in alfalfa stands remain unexplored. The aim of this study was to identify the linkages of rhizosphere and root metabolites, particularly autotoxins and prebiotics, to alfalfa decline under continuous cropping. We performed liquid chromatography–mass spectrometry for non-targeted metabolomic profiling of rhizosphere soils and alfalfa roots in 2- and 6-year-old stands. Differentially abundant metabolites that responded to stand age and associated metabolic pathways were identified. Compared with bulk soils, rhizosphere soils were enriched with more triterpenoid saponins (e.g., medicagenic acid glycosides), which showed inhibitory effects on seed germination and seedling growth. These autotoxic metabolites were accumulated in the old stand age, and their relative abundances were negatively correlated with plant growth, yield, and quality traits, as well as soil total nitrogen and alkali-hydrolyzable nitrogen concentrations. In contrast, prebiotic metabolites, represented by glycerolipids (e.g., glycerophosphocholine) and fatty acyls (e.g., colnelenic acid), were depleted in rhizosphere soils in the old stand. The relative abundances of glycerolipids and fatty acyls were positively correlated with plant traits and soil available phosphorus and alkali-hydrolyzable nitrogen concentrations. Age-induced changes in the rhizosphere metabolome mirrored the reprogramming patterns of root metabolome. The pathways of terpenoid backbone biosynthesis and plant hormone signal transduction, as well as metabolism of galactose, glycerophospholipid, and ɑ-linolenic acid in alfalfa roots were affected by stand age. The upregulation of terpenoid backbone biosynthesis in alfalfa roots of old plants, which stimulated triterpenoid saponin biosynthesis and exudation. Rhizosphere accumulation of autotoxins was accompanied by depletion of prebiotics, leading to soil degradation and exacerbating alfalfa decline. This research aids in the development of prebiotics to prevent and manage continuous cropping obstacles in alfalfa.

## 1 Introduction

Alfalfa (*Medicago sativa* L.) is a perennial leguminous forage widely grown for hay and silage in North America, Europe, and Asia. Continuous cropping leads to a decline in both yield and nutritional quality, with the best growth performance observed in 2-year-old stands. Continuous cropping is the practice of planting the same crop in the same field over multiple growing seasons without rotation, often resulting in various challenges known as continuous cropping obstacles (Jiao et al., 2024). Alfalfa stands aged 4 years and older suffer dramatic yield loss and quality reduction due to inferior plant growth, poor root development, and diminished disease resistance (Rong et al., 2016). This phenomenon is a typical continuous cropping obstacle of alfalfa (Wang et al., 2022a), and this problem is considered a long-term bottleneck restricting the regional development of the alfalfa industry in the loess hilly region of northwestern China (Ma et al., 2024). Effective preventive strategies need to be designed and implemented to avoid devastating economic consequences caused by alfalfa decline under continuous cropping (Sun and Li, 2019).

The decline in yield and quality of many crops after long-term continuous cropping is at least partly attributed to rhizosphere accumulation of allelopathic autotoxins (Xiong et al., 2024). For example, vanillin inhibits potato (*Solanum tuberosum* L.) growth by lowering root auxin content and adventitious root number (Ma et al., 2023). Additionally, cinnamic acid stimulates the pathogenicity of Fusarium oxysporum and weakens the physio-biochemical resistance of plants, hindering faba bean (*Vicia faba* L.) growth (Guo et al., 2020). In the black soil region of northeastern China, continuous cropping of alfalfa leads to rhizosphere accumulation of coumaric acid and enrichment of potentially harmful fungi (Wang et al., 2022a). In sharp contrast to the fertile and moist black soils, the highly erodible loessial soils of the loess hilly region in northwestern China have low moisture and organic matter content (Han and Li, 2018). This leads to the following questions: What allelopathic autotoxins are responsible for alfalfa decline in the rainfed agricultural area of the loess hilly region, and are these rhizosphere metabolites exuded from alfalfa roots?

The niche adjacent to plant roots is defined as the rhizosphere, which is a unique soil zone with the highest levels of root exudates and root-recruited microbes (Brink, 2016). Rhizosphere metabolites primarily consist of root exudates, along with small molecules generated by microbial metabolism. The composition and abundance of rhizosphere metabolites are determined by plant species and soil environmental conditions (Sugiyama, 2019). Rhizosphere metabolites modulate root growth and plant health by participating in multiple biological processes, such as belowground chemical communication, root–microbe interactions, and plant nutrient stress (Massalha et al., 2017). However, no study has elucidated age-induced metabolic reprogramming that underpins alfalfa decline.

The aim of this study was to unravel the chemical mechanism of alfalfa decline in the loess hilly region of northwestern China. Rhizosphere-enriched metabolites were identified by comparing metabolite profiles between rhizosphere soils of different stand ages and bulk soils. Potential autotoxins and prebiotics responsible for alfalfa decline were determined on the basis of the quantitative changes of age-related metabolites and their relationship to plant traits. The interactions between rhizosphere and root metabolites were analyzed to decipher plant-soil crosstalk mediating alfalfa decline. The results could provide novel mechanistic insight into alfalfa decline and unlock new possibilities for early detection and prevention.

## 2 Materials and methods

### 2.1 Study area and site selection

The study was conducted in Pengyang County (106° 38′E, 35° 4l′N), which represents a rainfed agricultural area in the loess hilly region of southern Ningxia, China. This area is located in the central hilly gully region of the Loess Plateau with a typical temperate semi-arid continental monsoon climate. The mean annual temperature ranges from 7.4°C to 8.5°C, and the mean annual precipitation is 429.8 mm. The major soil type is classified as loessial soil (Entisol). Because the national policy of returning farmland to forest and grassland was implemented in 2003, alfalfa has been widely established in the study area. The experiment began in 2017 and ended in 2023. We selected two field plots (48 m × 12 m each) with the same elevation and spaced ~100 m apart. Alfalfa was grown in one plot in April 2017 (after 2 years of maize cropping) and in the other plot in April 2021 (after 6 years of maize cropping). Alfalfa seeds of the cultivar “Gannong No. 4” were provided by Shanggu Agriculture and Animal Husbandry Development Co. Ltd. (Ningxia, China) and drill sown at a rate of 21 kg/ha, with a row spacing of 40 cm. The plots were managed under the same practices, including annual harvest time and fertilization rate. Sampling was carried out at the first full-bloom stage of alfalfa in May 2023.

### 2.2 Sample collection

Six 1 m × 1 m quadrats were randomly selected in each plot, and six alfalfa plants were randomly selected within each quadrat. A soil profile of ~30 cm depth was dug up using a shovel within 50 cm from the plant stem to expose the roots. A soil knife was used to collect the soil loosely attached to the roots, and fine lateral roots were gently shaken to collect the fallen soil; these soils were combined and stored at 4°C until used for chemical analysis. The remaining soil adhering to the roots (i.e., rhizosphere soil) was collected using a sterile bristle brush and passed through a 20-mesh sieve after removing impurities with sterilized forceps. The rhizosphere soils of six plants from the same quadrat were thoroughly mixed to obtain one composite sample. Then, 15.00-g soil samples were sealed in sterile centrifuge tubes and immediately frozen at −80°C for metabolomic analysis. Additionally, six bare patches without alfalfa or weed growth were selected in each quadrat. After removing ~1-cm-thick topsoil, bulk soil was collected from a depth of 30 cm with an earth auger. The bulk soils from each quadrat were mixed well, with subsamples separately processed for physicochemical and metabolomic analyses.

After rhizosphere soil sampling, the roots were flushed with sterile water, and the fine lateral roots were cut off using sterilized scissors. Then, the cortical part of the main root was stripped with a sterilized knife, with 15.00 g of samples sealed in centrifuge tubes and stored at −80°C for metabolomic analysis. The roots of six plants within each quadrat were mixed into one composite sample, resulting in a total of six root samples per plot.

### 2.3 Determination of soil properties and plant traits

Soil samples were air-dried, ground, and passed through a 20-mesh sieve before chemical analysis. The potassium dichromate volumetric method was used to determine soil organic matter (Wu and Chen, 2016). For quantification of soil total and alkali-hydrolyzable nitrogen, semi-micro Kjeldahl and alkali hydrolysis methods were respectively used (Zhang et al., 2015). Soil total phosphorus and potassium were analyzed by molybdenum-antimony anti-colorimetric assay and flame photometry, respectively, with samples fused using sodium hydroxide. Ultraviolet spectrophotometry was conducted to analyze soil available phosphorus in sodium bicarbonate extracts (Wang et al., 2009). Flame photometry was adopted to determine soil available potassium in ammonium acetate extracts (Dai and Li, 1997). Soil pH measurement was carried out in 1:2 (w/v) slurries using a digital pH meter (PHST-5; Merweather Biotechnology Co., Ltd., Shanghai, China), with total salt determined by conductometry (Gu et al., 2023).

The shoots of alfalfa plants remaining in the six quadrats of each plot were harvested and used for yield and quality assessment. All plant samples were sealed in plastic bags and brought back to the laboratory. The fresh weight per plant and the total fresh weight of all plants from each quadrat were determined using an electronic scale (accuracy: 0.01 g). Biomass yield was calculated by dividing the total fresh weight of all plants per quadrat by the quadrat area. Plant height was measured using a ruler, and stem thickness was obtained using digital Vernier calipers. The stems and leaves were separated, deactivated at 105°C for 1 h, and oven-dried at 70°C for 48 h until constant weight. The dry weight of stems and leaves was determined to calculate the leaf-to-stem ratio. After deactivation (105°C, 24 h), shoot samples were analyzed for crude protein using the procedure given by AOAC International (1995). Filter bags were used for crude fiber analysis (Yang and Chen, 2017), with neutral and acid detergent fiber determined using a fiber analyzer (A200i; ANKOM, Macedon, NY, USA) based on the method of Soest et al. (1991). Crude fat (ether extract) was determined using an automatic fat analyzer (AXT15i; ANKOM) as described by Li et al. (2022).

### 2.4 Non-targeted metabolomics

The procedure reported by Fu et al. (2023) was used for non-targeted metabolomics. Briefly, metabolites were extracted using pre-cooled MeOH: ACN: H_2_O solution (v:v:v = 2:2:1) from rhizosphere soils and alfalfa roots for ultrahigh–performance liquid chromatography–quadrupole time-of-flight-mass spectrometry (UHPLC-QTOF-MS), with an injection volume of 10 mL. The separation was performed on an HSS T3 C18 column (100 mm × 2.1 mm × 1.8 mm; Waters Corp., Milford, MA, USA) with the temperature maintained at 50°C. The gradient elution program comprised mobile phase A (0.1% formic acid in water) and mobile phase B (0.1% formic acid in ACN) at a flow rate of 200 µL/min. The gradient was as follows: 0–2 min, 100% A; 2–11 min, 0%–100% B; 11–13 min, 100% B; 13–15 min, 0%–100% A.

The Q-TOF mass spectrometer was operated in positive and negative ion modes under the following conditions: electrospray ionization source temperature at 120°C, desorption temperature at 450°C, desorption gas flow rate of 800 L/h, cone gas flow rate of 50 L/h, TOF mass range of 50–1,200 Da, and scan time of 0.2 s. Metabolite profiles were acquired using an ACQUITY UHPLC system (Waters Corp.) coupled with a Triple TOF 5600 system (AB SCIEX, Framingham, MA, USA). Quality control samples were injected every six samples throughout the analysis to assess the stability of the analysis. The raw data were provided by Allwegene (Beijing, China), and metabolites were identified primarily on the basis of retention time (RT) mass-to-charge ratio (m/z) pairs and tandem mass spectrometry spectra, using HMDB (https://hmdb.ca/), LIPID MAPS (https://lipidmaps.org/), and a self-constructed database. The data matrix, including three-dimensional datasets of m/z, RT peaks, and intensities, was exported as an Excel file for further analysis.

### 2.5 Seed germination assay

The toxicity of rhizosphere-enriched metabolites represented by triterpenoid saponins was tested in Petri dishes. Medicagenic acid (purity ≥ 98%) and bayogenin (purity ≥ 98%) were purchased from Tianzhi Biotechnology Co., Ltd. (Wuhan, Hubei Province, China). The metabolites were dissolved in dimethyl sulfoxide to formulate working solutions at gradient concentrations of 1 mM, 10 mM, 100 µM, 50 µM, 25 µM, 12.5 µM, 6.25 µM, 3.125 µM, 1.562 µM, 0.781 µM, 0.39 µM, and 0 µM. Alfalfa seeds were disinfected with 75% ethanol for 2 min and then immersed in the metabolite solutions for 12 h. After that, 20 seeds were placed on sterile filter paper in a 90-mm-diameter Petri dish and incubated in a 28°C incubator under dark conditions. Seed germination rate (%) was calculated by dividing the number of germinated seeds at 2 days by the total number of seeds, and the growth of seedlings was observed at 5 days.

The toxicity of root extracts was also tested using Petri dish assay. After collecting rhizosphere soil and root samples, the roots of remaining alfalfa plants in each quadrat were dug up. Soil attached to the root surface was removed, and the roots were then dried and crushed. A 9.00-g sample of crushed roots was extracted with 500 mL of pure water for 12 h, followed by 30 min of boiling. Then, the roots were discarded, and the extract was continuously boiled until reaching a final volume of 50 mL. The extract was centrifuged at 845g for 5 min, and the supernatant was collected to prepare working solutions at serial dilutions of 0-, 5-, and 50-fold. The root extract solutions (5 mL each) were added to sterile filter paper in Petri dishes that contained 20 alfalfa seeds. Seed germination rate was analyzed after 2 days of incubation.

### 2.6 Data processing and analysis

Metabolomic raw data were collected using MassLynx v4.2 and processed using Progenesis QI v3.0 (Waters Corp., Milford, CT, USA). Annotation and identification of metabolites were performed using the METLIN database (Waters Corp.) and an in-house MS2 database (Allwegene.DB) (Yu et al., 2024). To distinguish metabolic differences between sample groups, orthogonal partial least squares discriminant analysis (OPLS-DA) was carried out using the “ropls” package in R v3.3.2 (Boccard and Rutledge, 2013). Differentially abundant metabolites (DAMs) were visualized by volcano plots using the R “ggplot2” package (Valero-Mora, 2010). The selection of DAMs was based on the P-value of Student’s t-test < 0.05 and the variable importance in projection (VIP) > 1 for the first principal component of the OPLS-DA model. Univariate analysis (one-way analysis variance) of metabolomic data was performed using an online resource (http://www.metaboanalyst.ca/) (Miska et al., 2021). Biological pathway analysis was performed on metabolomic data using MetaboAnalyst v5.0, with the pathway impact value > 0.05. Spearman’s correlation analysis was performed to determine the relationship between major DAMs, as well as among DAMs, plant traits, and soil properties. The results of plant traits and soil properties are presented as the mean ± standard deviation (n = 6). IBM SPSS v27.0 (IBM Corp., Armonk, NY, USA) was used to perform one-way analysis of variance, followed by the least significant difference test for multiple comparisons. Bar charts were produced using Sigmaplot v15.0 (Systat Software Inc., San Jose, CA, USA).

## 3 Results

### 3.1 Plant traits and soil nutrients

**Figure 1:**
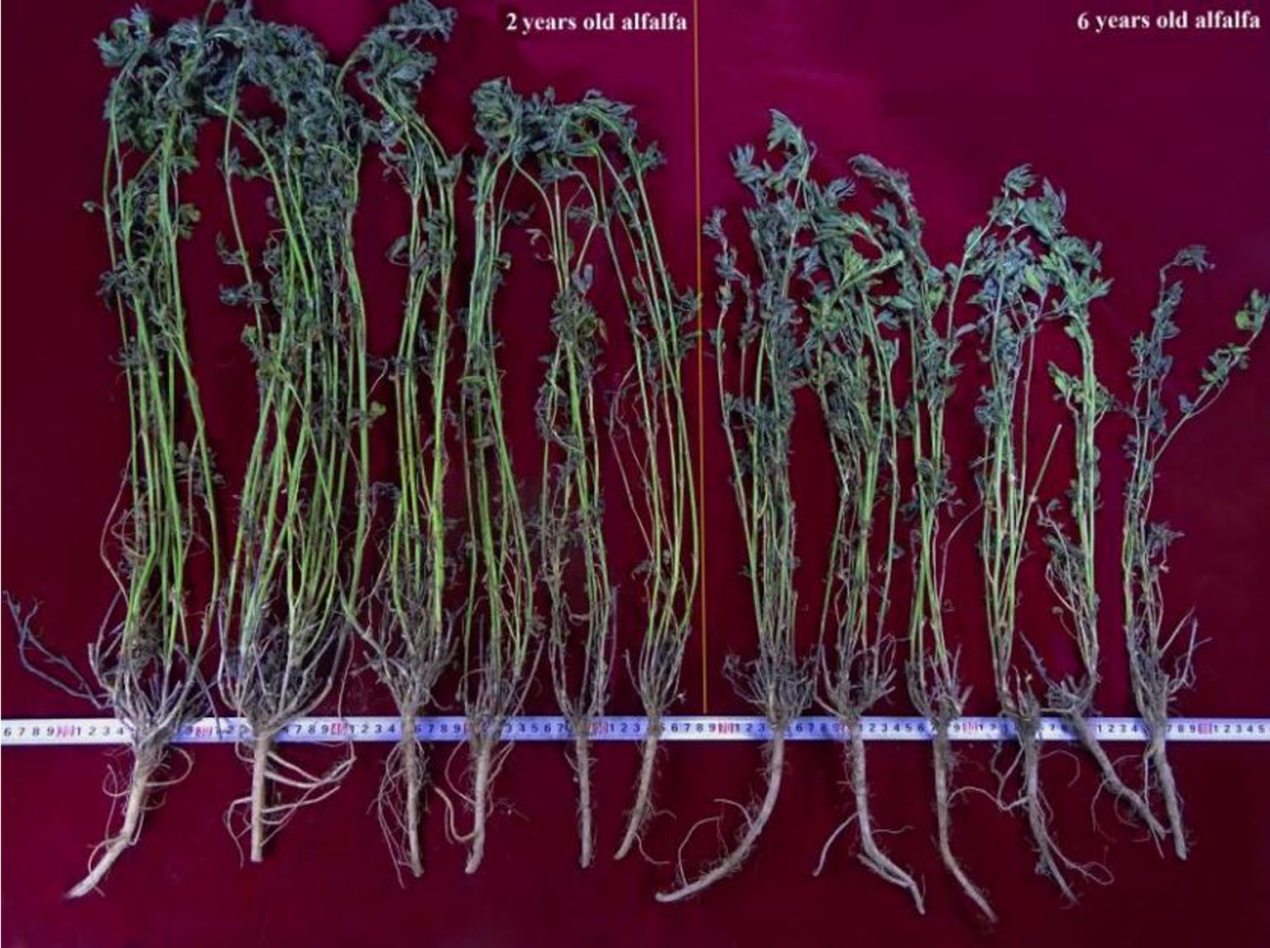
Photograph of young and old alfalfa plants.

Inferior plant growth was observed in the 6-year-old stand compared to that in the 2-year-old stand (**Figure 1**). This was manifested by lower stem height (by 10.3%), shoot fresh weight (by 48.6%), leaf-to-stem ratio (by 26.5%), and biomass yield (by 41.6%) of the old stand than the young stand (*P* < 0.05). Plant nutritional quality also decreased with increasing stand age in terms of shoot crude protein and fiber contents (by 13.3% and 10.0%, respectively; *P* < 0.05). Soil nutrients showed distinct differences between rhizosphere and bulk soils of the same stand age (**Table 1**). The total nitrogen, alkali-hydrolyzable nitrogen, and available phosphorus concentrations in 2R soils were 7.4% (*P* < 0.05), 14.6%, and 41.4% (*P* < 0.05) higher than those of 2C soils. However, these soil properties were 24.1%, 33.8%, and 13.4% lower in 6R soils compared to those in 6C soils (*P* < 0.05). These results indicate that 6-year-old alfalfa has suffered from severe continuous cropping obstacles.

**Table 1:** Nutritional quality and biomass yield of alfalfa and soil nutrient concentrations in differently aged stands.

Group	Crude protein (%)	Crud fat (%)	Crude fiber (%)	Neutral detergent fiber (%)	Acid detergent fiber (%)	Stem height (cm)	Shoot fresh weight (g/plant)	Leaf-to-stem ratio (%)	Yield (kg/m^2^)
Young stand	19.64 ± 2.13a	1.38 ± 0.52a	28.91 ± 3.24a	34.69 ± 4.67a	29.67 ± 3.48a	71.5 ± 10a	14.0 ± 6.3a	3.4 ± 1.2a	1.85 ± 0.54a
Old stand	17.02 ± 1.20b	1.43 ± 0.33a	26.02 ± 2.95b	33.85 ± 5.02a	28.26 ± 0.15a	64.1 ± 12b	7.2 ± 5.4b	2.5 ± 0.6b	1.08 ± 0.60b
Percent change (%)	−13.3	3.6	−10.0	−2.4	−4.8	−10.3	−48.6	−26.5	−41.6
Group	Total N (g/kg)	Total P (g/kg)	Total K (g/kg)	Alkali-hydrolyzable N (mg/kg)	Available K (mg/kg)	Available P (mg/kg)	Organic matter (g/kg)	Total salt (g/kg)	pH
2C	0.68 ± 0.22b	0.61 ± 0.12a	13.54 ± 1.35a	27.41 ± 0.10a	56.42 ± 1.25b	9.79 ± 2.88b	11.44 ± 3.45ab	0.2 ± 0.05a	8.43 ± 0.11a
2R	0.73 ± 0.41a	0.64 ± 0.08a	13.76 ± 2.03a	31.4 ± 2.56a	56.21 ± 3.66b	13.84 ± 0.69a	10.67 ± 2.36b	0.25 ± 0.08a	8.28 ± 0.05a
Percent change (%)	7.4	4.9	1.6	14.6	0.4	41.4	-6.7	0.25	−1.8
6C	0.87 ± 0.19a	0.67 ± 0.10a	14.6 ± 0.95a	36.97 ± 1.02a	85.12 ± 2.39a	7.75 ± 2.75b	13.87 ± 0.29a	0.22 ± 0.01a	8.39 ± 0.08a
6R	0.66 ± 0.31b	0.64 ± 0.25a	13.78 ± 1.48a	24.47 ± 3.05b	75.17 ± 2.16a	6.71 ± 1.45b	10.68 ± 2.88b	0.22 ± 0.04a	8.35 ± 0.15a
Percent change (%)	−24.1	−4.5	−5.6	−33.8	−11.7	−13.4	−3.19	0	−0.5

Values are the mean ± standard deviation of six replicates. Significant differences between groups are indicated by different lowercase letters in the same column (*P* < 0.05, Student’s t-test). 2C and 2R are bulk and rhizosphere soils from the 2-year-old stand, respectively; 6C and 6R are bulk and rhizosphere soils from the 6-year-old stand, respectively.

### 3.2 Rhizosphere-enriched metabolites

**Figure 2:**
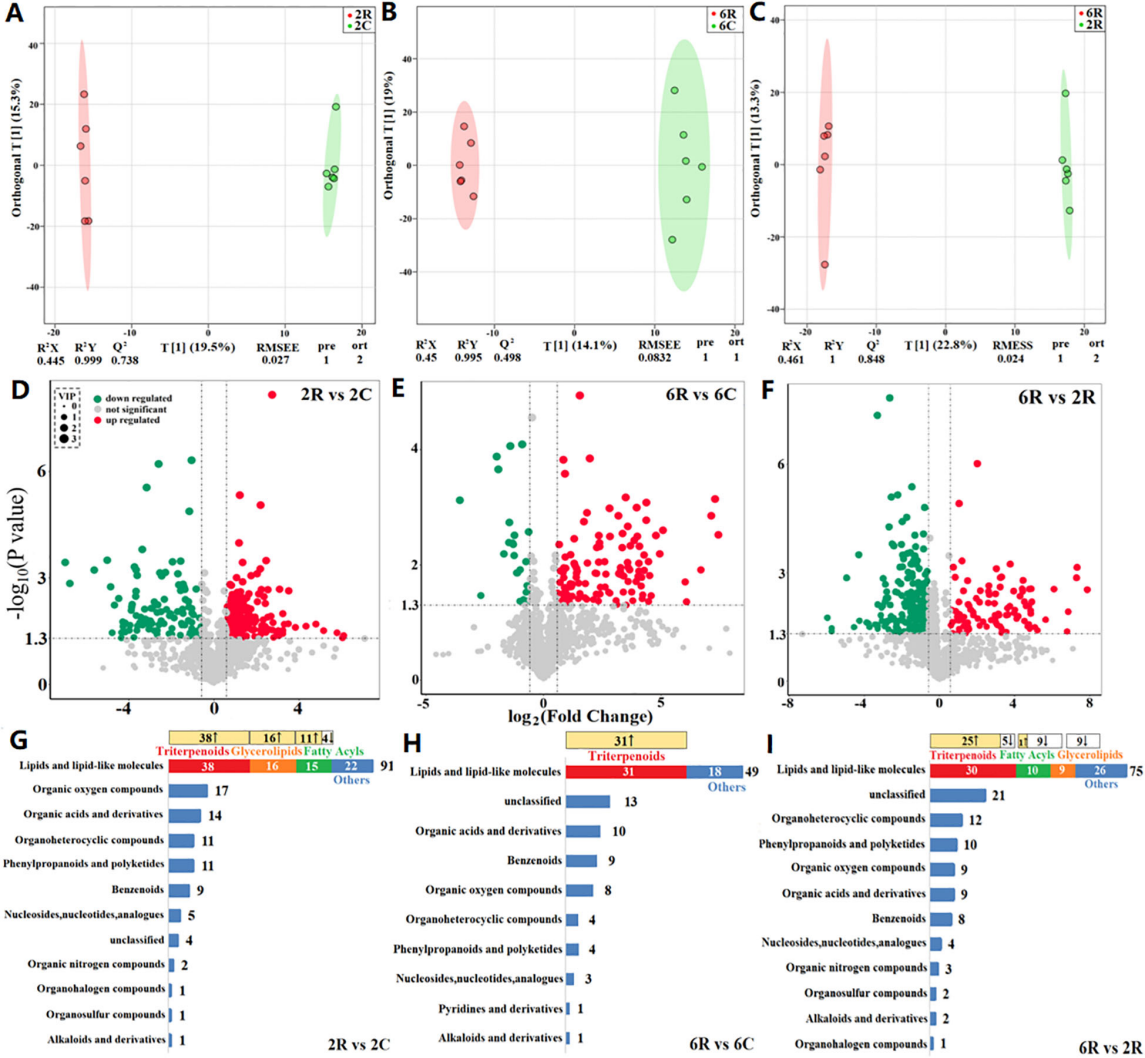
Metabolomic profiling of rhizosphere and bulk soils from alfalfa stands of different ages. **(A–C)** Orthogonal partial least squares discriminant analysis (OPLS-DA) score plots based on non-targeted metabolomic data. 6R and 6C represent rhizosphere and bulk soils from the 6-year-old stand, respectively; 2R and 2C represent rhizosphere and bulk soils from the 2-year-old stand, respectively. R^2^X and R^2^Y are the fraction of the variance of the X and Y matrices explained by the model, respectively; Q^2^ is the predictive squared correlation coefficient obtained by cross-validation; RMSEE is the root mean squared error; pre is the number of predictive components; and ort is the number of orthogonal components. The model parameters closer to 1 indicate a better model fit to the data. **(D–F)** Volcano plots of differentially abundant metabolites. **(G–I)** Composition of differentially abundant metabolites. The change in the relative abundance of metabolites that comprised the highest proportions of lipids and lipid-like molecules is shown at the top (↑ upregulation and ↓ downregulation).

Using UHPLC-QTOF-MS, we identified a total of 1,451 metabolites across 24 soil samples, with 905 metabolites annotated. OPLS-DA models revealed a distinct separation between rhizosphere and bulk soils of the same stand age (6 years: 6R vs. 6C, **Figure 2A**; and 2 years: 2R vs. 2C, **Figure 2B**), as well as between rhizosphere soils of different stand ages (6R vs. 2R, **Figure 2C**). This is indicative of prominent differences in metabolite profiles among soil samples. The DAMs between rhizosphere and bulk soils of the same stand age, as well as those between rhizosphere soils of different stand ages, were predominantly lipids and lipid-like molecules. Of these, triterpenoids, fatty acyls, and glycerolipids were most abundant (**Figures 2G–I**).

To ascertain unique metabolites in the rhizosphere of alfalfa and their response patterns to stand age, we identified DAMs with higher relative abundances in rhizosphere soils than in bulk soils, i.e., rhizosphere-enriched metabolites. A total of 167 DAMs were identified in 2R vs. 2C soils, which mainly comprised 12 classes, including lipids and lipid-like molecules, as well as organic oxygen compounds (**Figure 2G**). Of these, 127 DAMs were upregulated in the rhizosphere (**Figure 2D**), and these rhizosphere-enriched metabolites primarily consisted of triterpenoids, glycerolipids, and fatty acyls (lipids and lipid-like molecules; **Figure 2G**). Additionally, 102 DAMs were identified in 6R vs. 6C soils, mainly including lipids and lipid-like molecules, as well as organic acids and derivatives (**Figure 2H**). Among them, 85 DAMs were upregulated in the rhizosphere (**Figure 2E**), and these rhizosphere-enriched metabolites were predominantly triterpenoids (**Figure 2H**). Triterpenoids (triterpenoid saponins) showed the highest relative abundance among the rhizosphere-enriched metabolites. The total abundance of these triterpenoid saponins (19 compounds) in 6R and 2R soils was 25- and 14-fold higher than that of 6C and 2C soils, respectively (**Table 2**).

**Table 2:** Relative abundance (×10^3^) of triterpenoid saponins in rhizosphere and bulk soils from alfalfa stands of different ages.

No.	Metabolite	6R	6C	Fold change	2R	2C	Fold change
1	Medicagenic acid 3-O-triglucoside	353	2	177^**^	75	2	38^**^
2	3-((3’-Malonyl)Xyl)-28-Glu Bayogenin	149	1	149^*^	38	1	38^**^
3	Medicoside J	51	1	51^*^	15	0	–
4	Medicagenic acid beta-maltoside	898	14	64^*^	366	18	20^*^
5	3-(Rha(1-2)Glu(1-2)Glu-28-Glu Hederagenin	32	1	32^**^	19	1	19^*^
6	Medicoside I	181	6	30^**^	80	6	13^**^
7	3-Ara-28-Glu Hederagenin	755	31	24^*^	618	22	28^*^
8	Hex-hex-hex-Pen-Bayogenin	24	1	24^**^	11	1	11^**^
9	Hex-hex-hexA-Bayogenin	109	6	18^*^	77	4	19^*^
10	Medicoside H	62	4	16^*^	27	2	14^**^
11	Hederagenin 3-O-arabinoside II	21	2	11^**^	15	2	8^*^
12	Soyasaponin IV	24	2	12^**^	10	2	5^**^
13	Medicoside E	67	7	10^*^	29	2	15^**^
14	Licoricesaponin D3	58	6	10^*^	29	3	10^*^
15	Hex-hexA-dhex Bayogenin	240	26	9^**^	202	25	8^*^
16	Hoduloside IV	17	1	17^**^	8	1	8^**^
17	Betavulgaroside IV	82	2	41^**^	14	4	4^*^
18	3-Epipapyriferic acid	70	8	9^*^	48	20	2^*^
19	Calenduloside H methyl ester	19	7	3^**^	16	7	2^*^
Sum		3212	128	25	1697	123	14

6R and 6C represent rhizosphere and bulk soils from the 6-year-old stand, respectively; 2R and 2C represent rhizosphere and bulk soils from the 2-year-old stand, respectively. *P < 0.05 and **P < 0.01 (Student’s t-test; *n =* 6).

### 3.3 Age-related rhizosphere metabolites

**Figure 3:**
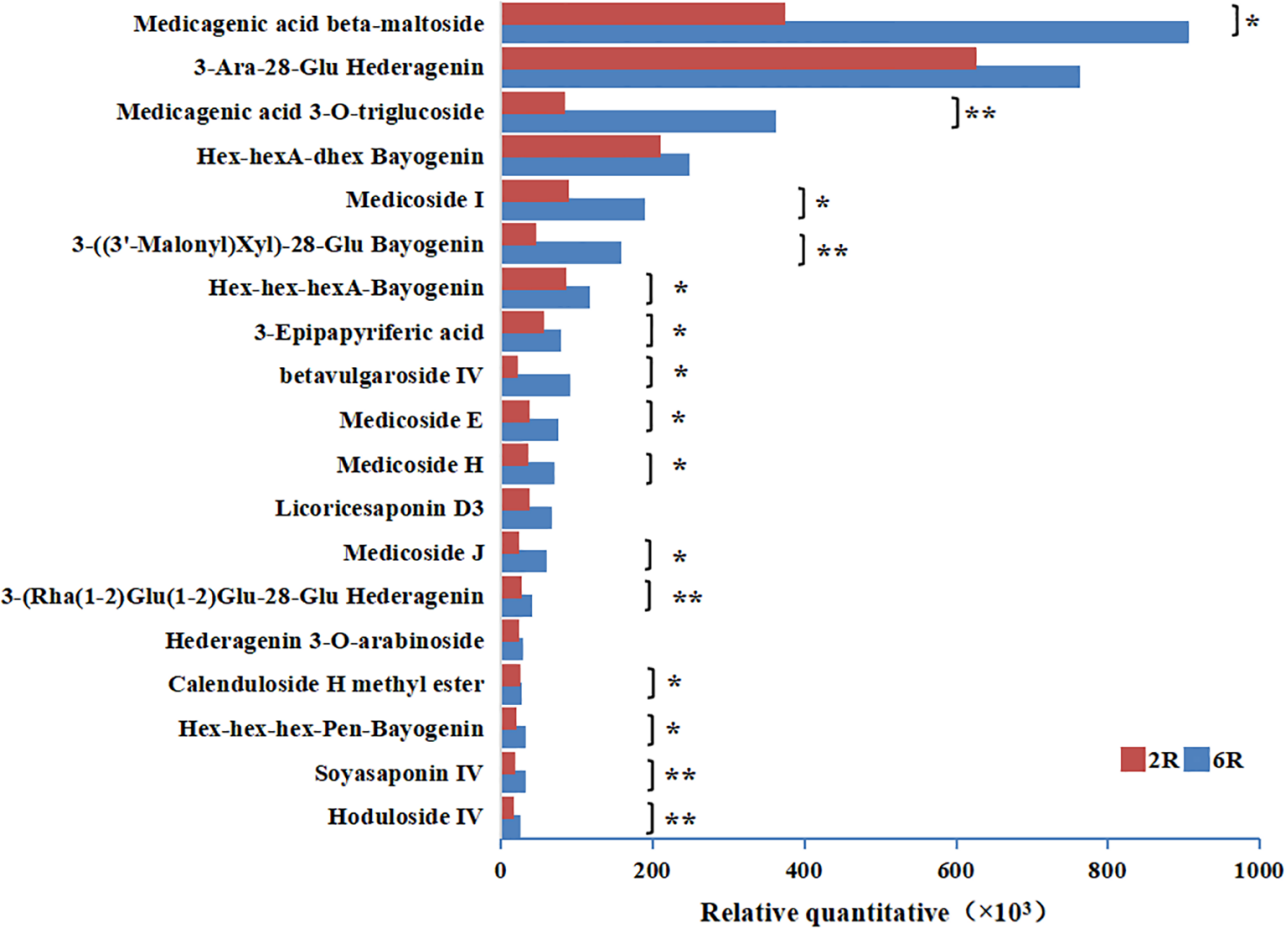
Changes in the relative abundances (×10^3^) of triterpenoid saponins in rhizosphere soils from alfalfa stands aged 2 years (2R) and 6 years (6R). *P < 0.05 and **P < 0.01 (Student’s t-test; *n =* 6).

To elucidate the role of stand age in alfalfa decline, we identified DAMs with different relative abundances between rhizosphere soils of different stand ages, i.e., age-related rhizosphere metabolites. A total of 157 DAMs were identified in 6R vs. 2R soils. These metabolites mainly comprised 12 classes, including lipids and lipid-like molecules, as well as organoheterocyclic compounds (**Figure 2I**). Among them, 99 DAMs were downregulated in 6R soils (**Figure 2F**), principally represented by fatty acyls and glycolipids (lipids and lipid-like molecules), as well as pyrimidines and pyrimidine derivatives (organoheterocyclic compounds). The other 58 DAMs were upregulated in 6R soils (**Figure 2F**), predominantly triterpenoids (triterpenoid saponins; **Figure 2I**). Notably, 15 out of 19 triterpenoid saponins were accumulated significantly in rhizosphere soils with increasing stand age (*P* < 0.05; **Figure 3**).

**Figure 4:**
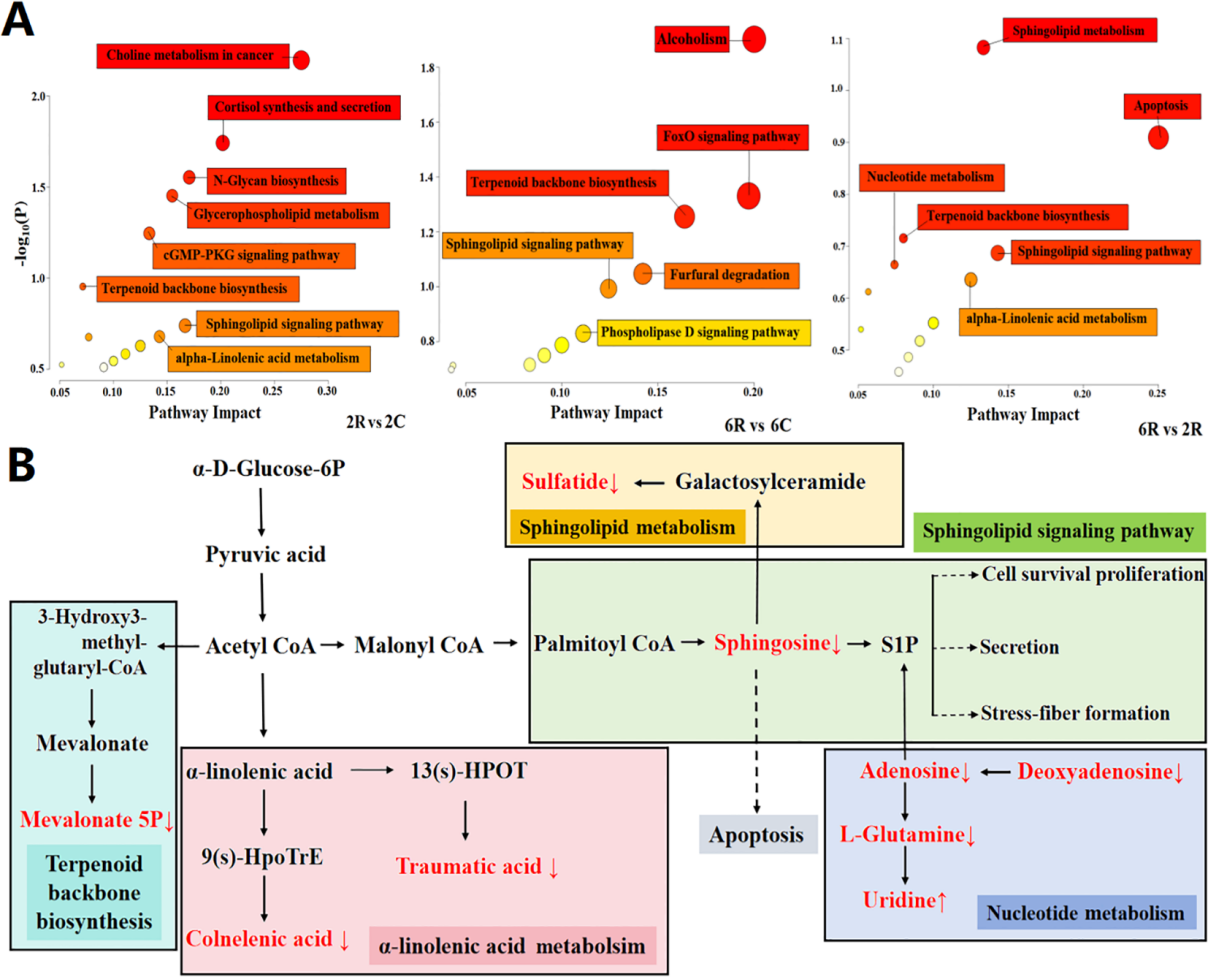
Metabolic pathways of age-related metabolites in the rhizosphere of alfalfa. **(A)** Enrichment of important metabolic pathways. 2R and 2C are rhizosphere and bulk soils from the 2-year-old stand, respectively; 6R and 6C are rhizosphere and bulk soils from the 6-year-old stand, respectively. Each bubble represents a metabolic pathway, with its size and color representing pathway enrichment and impact values, respectively. Log_10_(P) values are obtained by pathway enrichment analysis, and pathway impact values are obtained by pathway topological analysis. **(B)** Major metabolic pathways in 6R soils. Age-related metabolites are marked in red color (↑ upregulation and ↓ downregulation).

To identify age-induced changes in the rhizosphere metabolome, we performed pathway enrichment analysis of age-related rhizosphere metabolites and constructed a map of five metabolic pathways with the greatest impact factor. The age-related metabolites were primarily enriched in apoptosis, sphingolipid signaling pathway, sphingolipid metabolism, nucleotide metabolism, α-linolenic acid metabolism, and terpenoid backbone biosynthesis (**Figure 4A**). Next, we looked at the changes of major metabolic pathways in 6R soils compared to those in 2R soils (**Figure 4B**). Sphingosine, a key signal molecule in apoptosis, sphingolipid metabolism, and sphingolipid signaling pathways, was 0.6-fold downregulated in 6R soils (*P* < 0.001). The same pattern was observed for adenosine, a key signal molecule in the sphingolipid signaling pathway, which was 0.5-fold downregulated in 6R soils (*P* < 0.05). Deoxyguanosine and adenosine related to nucleotide metabolism were also downregulated in 6R soils, accompanied by upregulation of uridine (*P* < 0.05). Two fatty acyls related to α-linolenic acid metabolism, traumatic acid and colnelenic acid, were both 0.2-fold downregulated in 6R soils (*P* < 0.001), whereas mevalonate 5P involved in terpenoid backbone metabolism was 1.9-fold upregulated (*P* < 0.05; **Supplementary Table S1**). The metabolites depleted in rhizosphere soils of older stand age might be potential prebiotics beneficial for alfalfa growth, whereas the metabolites (triterpenoid saponins) enriched in rhizosphere soils might be potential autotoxins responsible for alfalfa decline.

### 3.4 Seed germination response to rhizosphere-enriched metabolites and root extracts

**Figure 5:**
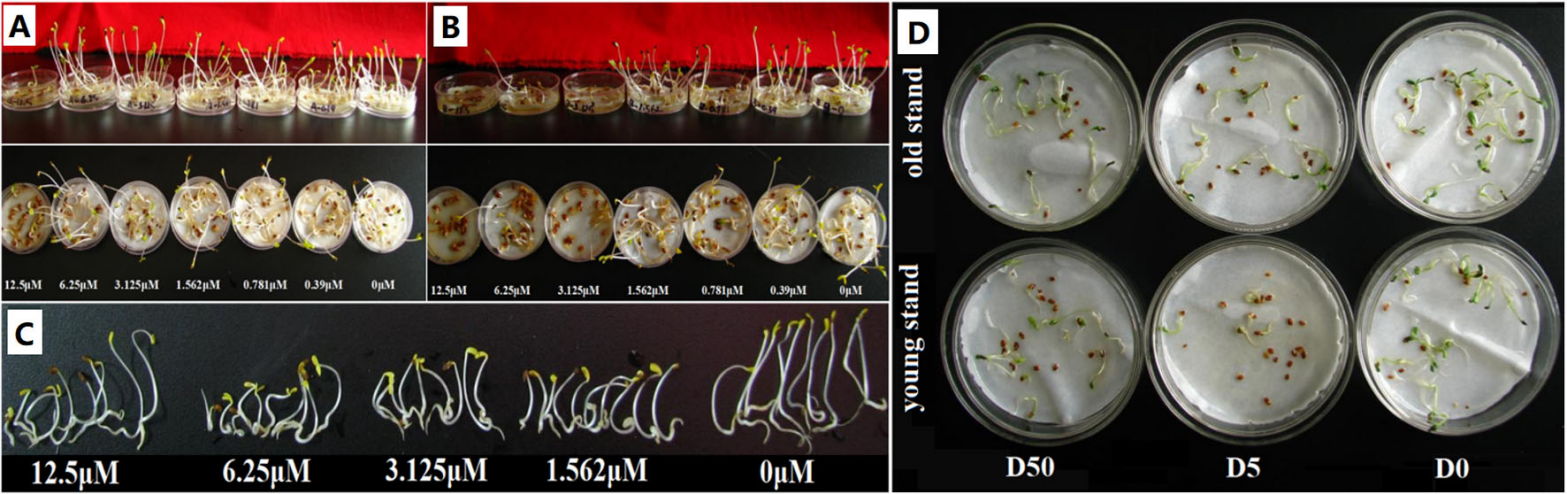
Effects of rhizosphere-enriched metabolites and root extracts on seed germination and seedling growth of alfalfa. **(A, B)** Seed germination after exposure to different concentrations of triterpenoid saponins (medicagenic acid and bayogenin). **(C)** Seedling growth under medicagenic acid treatment. **(D)** Seed germination in the presence of root extracts from 2- and 6-year-old alfalfa plants. D0, D5, and D50 represent 0-, 5-, and 50-fold serial dilutions, respectively.

To verify the potential toxicity of triterpenoid saponins in the rhizosphere to alfalfa growth, we carried out seed germination assay in Petri dishes. Compared to the control (seed germination rate 100%), exposure to 12.5 µM medicagenic acid reduced the average seed germination rate to 14% (**Figure 5A**, *P* < 0.05), and a low average seed germination rate of 4% was observed for 3.125 µM bayogenin treatment (**Figure 5B**, *P* < 0.05). Seedling growth was inhibited for 1.562 µM medicagenic acid treatment (**Figure 5C**), whereas the seed germination rate was 29% (**Figure 5A**). Both medicagenic acid and bayogenin prominently inhibited alfalfa seed germination and seedling growth. Further, we conducted seed germination assay with root extracts from 2- and 6-year-old alfalfa plants to preliminarily determine the source of autotoxic metabolites (e.g., triterpenoid saponins) in the rhizosphere. The average inhibition rate of seed germination by five-fold dilution of root extracts reached >50% (*P* < 0.05), and the root extract from old plants exhibited slightly greater inhibitory effect than that of young plants (**Figure 5D**). The results provide evidence for the allelopathic autotoxicity of triterpenoid saponins accumulated in the rhizosphere of old plants, and these autotoxins were most likely exuded from alfalfa roots.

### 3.5 Age-related root metabolites

**Figure 6:**
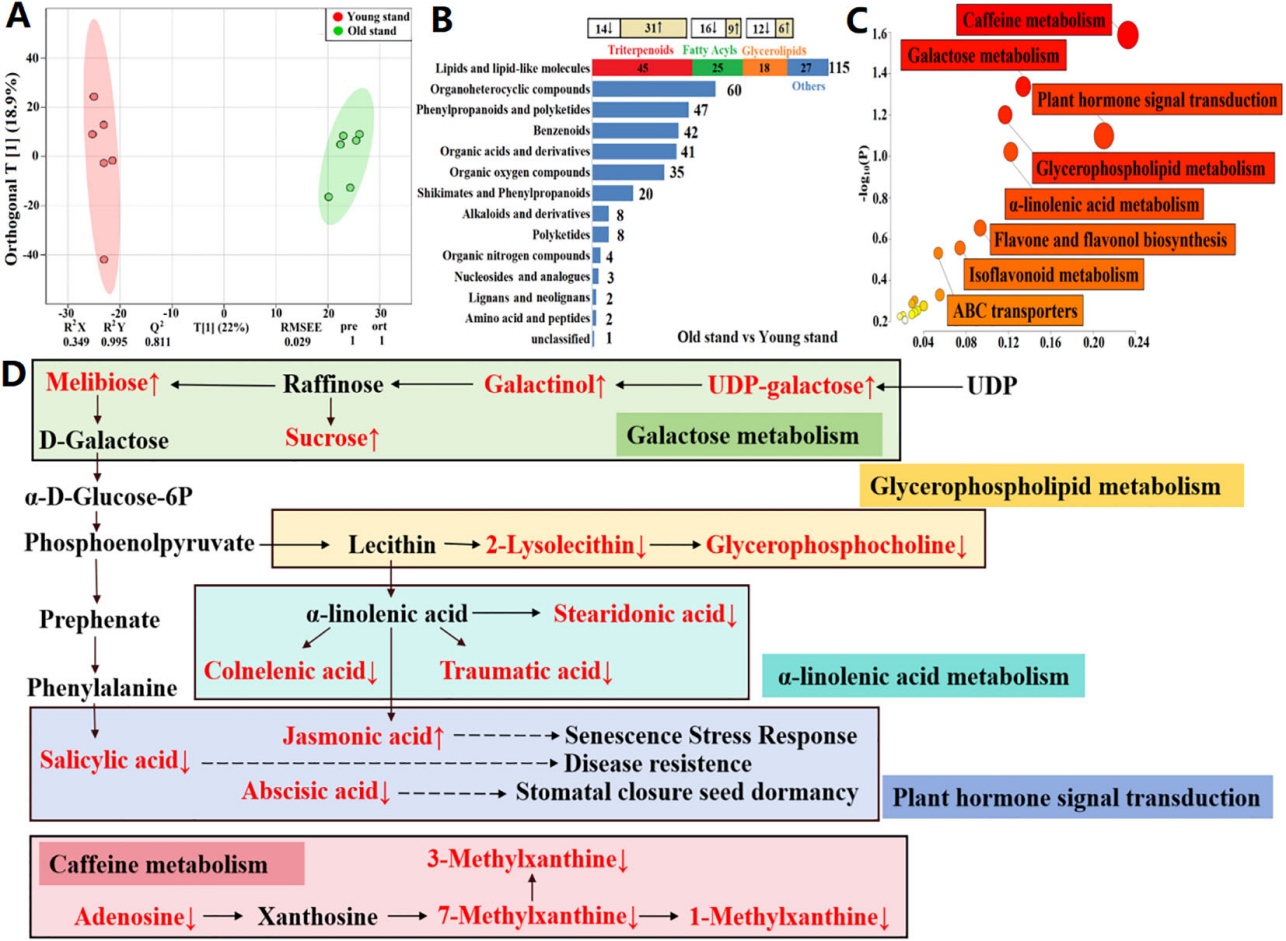
Metabolomic profiling and metabolic pathways of age-related metabolites in alfalfa roots. **(A)** Orthogonal partial least squares discriminant analysis (OPLS-DA) score plots based on non-targeted metabolomic data. R^2^X and R^2^Y are the fraction of the variance of the X and Y matrices explained by the model, respectively; Q^2^ is the predictive squared correlation coefficient obtained by cross-validation; RMSEE is the root mean squared error; pre is the number of predictive components; and ort is the number of orthogonal components. The model parameters closer to 1 indicate a better model fit to the data. **(B)** Composition of differentially abundant metabolites. The change in the relative abundance of metabolites that comprised the highest proportions of lipids and lipid-like molecules is shown at the top (↑ upregulation and ↓ downregulation). **(C)** Enrichment of important metabolic pathways. Each bubble represents a metabolic pathway, with its size and color representing pathway enrichment and impact values, respectively. Log_10_(P) values are obtained by pathway enrichment analysis, and pathway impact values are obtained by pathway topological analysis. **(D)** Major metabolic pathways in alfalfa roots of the old stand. Age-related metabolites are marked in red color (↑ upregulation and ↓ downregulation).

To verify the source of functional rhizosphere metabolites and plant-soil metabolic crosstalk, we conducted metabolomic profiling of alfalfa roots using UHPLC-QTOF-MS. A total of 2,763 metabolites were identified across 12 root samples, with 1,256 metabolites annotated. OPLS-DA models revealed a clear separation between root samples of different stand ages, emphasizing the differences among metabolite profiles (**Figure 6A**). There were 388 DAMs between root samples of different stand ages, i.e., age-related root metabolites, which were predominantly lipids and lipid-like molecules. Among them, triterpenoids, fatty acyls and glycerolipids were most abundant, similar to the composition of age-related rhizosphere metabolites (**Figure 2I**). In particular, triterpenoid saponins showed the highest relative abundance in roots of 6-year-old plants (**Figure 6B**), which mirrored the pattern in rhizosphere-enriched metabolites (**Figure 2I**). The total abundance of triterpenoid saponins (15 compounds) in root samples from the 6-year-old stand was two-fold higher than that of the 2-year-old stand, and most of the triterpenoid saponins showed significant differences in relative abundance between the two age groups (*P* < 0.05 or 0.01). Seven triterpenoid saponins detected in alfalfa roots, such as medicagenic acid, soyasaponin and medicoside, were also present in rhizosphere soils (**Table 3**). In contrast, glycerolipids, fatty acyls, and nucleotides were depleted in root samples from the 6-year-old stand compared to those from the 2-year-old stand (**Supplementary Table S2**), consistent with the pattern observed in rhizosphere metabolites (**Supplementary Table S1**). The results suggest that the potential autotoxins and probiotics in rhizosphere soils were mainly produced by alfalfa roots, and their biosynthesis was respectively upregulated and downregulated with increasing stand age.

**Table 3:** Relative abundance (×10^3^) of triterpenoid saponins in alfalfa roots of different stand ages.

No.	Metabolite	6-year-old stand	2-year-old stand	Fold change
1	※Soyasaponin V	227	122	2^*^
2	※Medicoside J	66	21	3^**^
3	※3-Epipapyriferic acid	327	64	5^**^
4	※Licoricesaponin E2	47	31	2
5	※Medicagenic acid	14	9	2
6	※Soyasaponin III	32	6	5^**^
7	※Betavulgaroside IV	18	7	3
8	Akebiasaponin D	1,530	276	6^**^
9	Cynarasaponin F	56	37	2^*^
10	Saikosaponin B2	192	47	4^*^
11	Phytolaccasaponin B	875	611	1
12	Asiaticoside	55	31	2^**^
13	Soyasaponin I	318	336	0.9
14	AZI	9	1	9^*^
15	AZII	6	3	2^*^
Sum		3,772	1,602	2^*^

*P < 0.05 and **P < 0.01 (Student’s t-test; *n =* 6). ※ indicates the metabolites also detected in rhizosphere soils.

To identify age-induced changes in the root metabolome, we performed pathway enrichment analysis of age-related root metabolites (**Figure 6C**) and constructed a map of five metabolic pathways with the highest impact factor (**Figure 6D**). The age-related metabolites were principally enriched in caffeine metabolism, plant hormone signal transduction, galactose metabolism, glycerophospholipid metabolism, and α-linolenic acid metabolism (**Figure 6C**). Melibiose, galactinol, and sucrose related to galactose metabolism were two-fold upregulated in alfalfa roots of the 6-year-old stand (*P* < 0.05), whereas two glycerolipids related to glycerophospholipid metabolism (glycerophosphocholine and 2-lysolecithin) were three-fold downregulated (*P* < 0.05). Additionally, three fatty acyles related to α-linolenic acid metabolism (stearidonic acid, traumatic acid, and colnelenic acid) were 0.5-, 0.5-, and 0.3-fold downregulated in alfalfa roots of the 6-year-old stand, respectively (*P* < 0.05), similar to the patterns of salicylic acid (0.3-fold) and abscisic acid (0.5-fold) involved in plant hormone signal transduction (*P* < 0.05; **Supplementary Table S2**).

### 3.6 Relationship of plant traits, soil nutrients, and age-related metabolites

**Figure 7:**
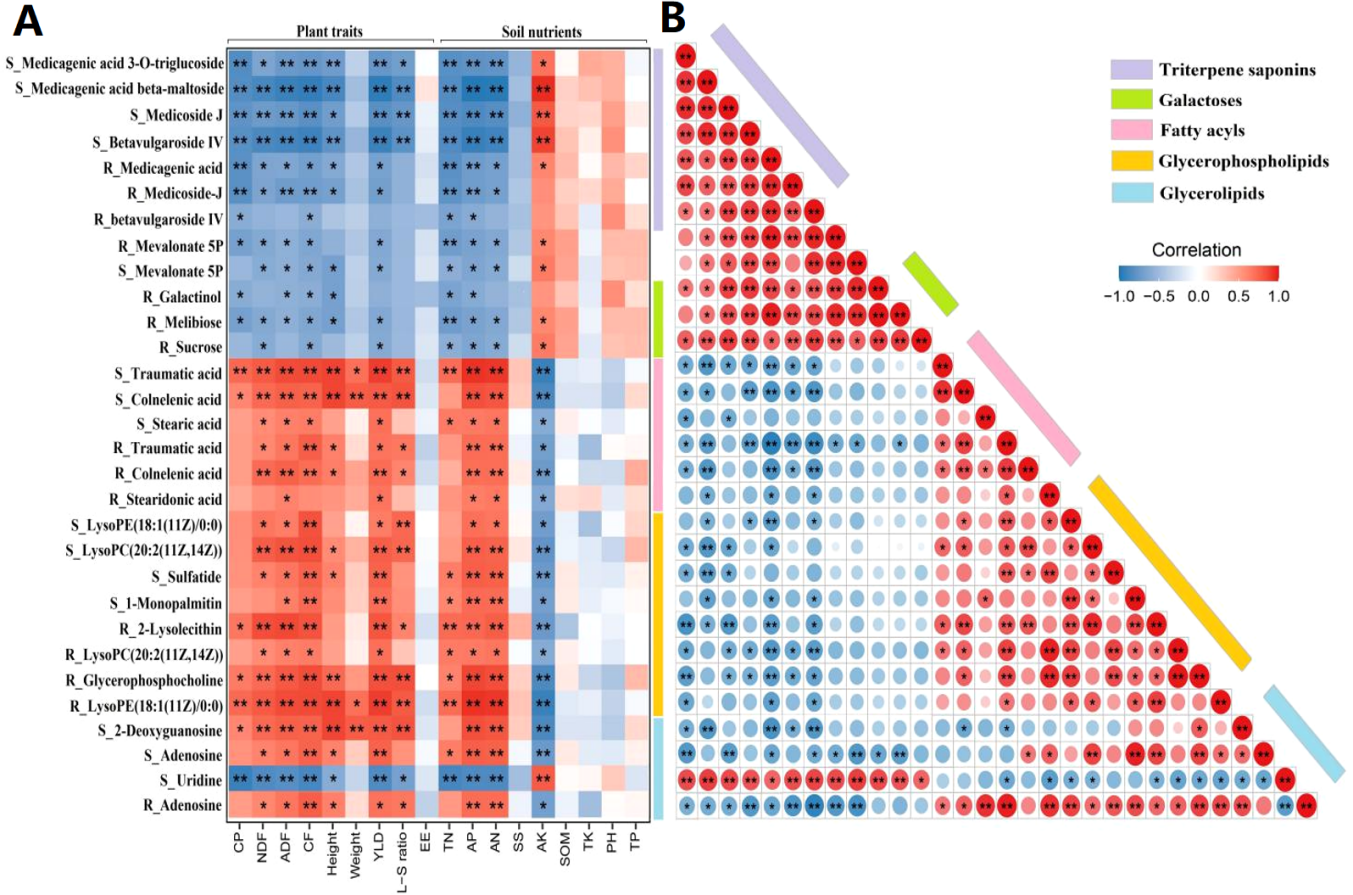
Relationship of plant traits, soil nutrients, and age-related metabolites. **(A)** Correlation matrix between rhizosphere/root metabolites and plant/soil variables. **(B)** Interactions between age-related metabolites in rhizosphere soils (S_) and alfalfa roots (R_). Positive and negative correlations are marked in red and blue colors, respectively; the color intensity is proportional to correlation strength. CP, crude protein; NDF, neutral detergent fiber; ADF, acid detergent fiber; CF, crude fiber; Height, plant height; Weight, shoot fresh weight; YLD, biomass yield; L-S ratio, leaf-to-stem ratio; EE, crude fat; TN, total nitrogen; AP, available phosphorus; AN, alkali-hydrolyzable nitrogen; SS, soil salt; AK, available potassium; SOM, soil organic matter; TK, total potassium; PH, power of hydrogen; and TP, total phosphorus. *P <0.05, **P <0.01.

To unravel the effects of rhizosphere and root metabolites on plant traits and soil nutrients, we calculated Spearman’s correlation coefficients between 18 plant and soil variables and the relative abundances of 30 age-related metabolites (15 rhizosphere metabolites and 15 root metabolites). Among the plant variables, shoot crude protein, crude fat, acid detergent fiber, and neutral detergent fiber contents, as well as plant height, leaf-to-stem ratio, and biomass yield were all significantly negatively correlated with triterpenoid saponins in rhizosphere soils and alfalfa roots (*P* < 0.01). Crude protein, crude fat, acid detergent fiber, and plant height were significantly negatively correlated with galactoses in alfalfa roots but not in rhizosphere soils (*P* < 0.01). With regard to soil variables, total nitrogen, alkali-hydrolyzable nitrogen, and available phosphorus concentrations were significantly negatively correlated with triterpenoid saponins and galactoses in rhizosphere soils and alfalfa roots (*P* < 0.05), whereas positive correlations emerged between these soil nutrients and fatty acyls, glycerophospholipids, and glyceroglycolipids in both cases (*P* < 0.05). Shoot neutral detergent fiber and crude fat contents as well as biomass yield were significantly positively correlated with deoxyguanosine and adenosine in rhizosphere soils (*P* < 0.05) and negatively correlated with uridine in rhizosphere soils (*P* < 0.05; **Figure 7A**).

Further, we unveiled intricate interactions between age-related rhizosphere and root metabolites based on Spearman’s correlation analysis of metabolite relative abundances (**Figure 7B**). Triterpenoid saponins in rhizosphere soils were positively correlated with these metabolites in alfalfa roots. Both sets of triterpenoid saponins were positively correlated with melibiose, galactinol, sucrose, uridine, and mevalonate 5P and negatively correlated with fatty acyls, glycerophospholipids, and glycerolipids in corresponding samples. Additionally, fatty acyls, glycerolipids, and nucleotides in rhizosphere soils were positively correlated with these metabolites in alfalfa roots. There were also positive correlations among fatty acyls, glycerolipids, deoxyguanosine, adenosine, and sphingosine in corresponding samples. The results indicate that the potential harmful metabolites that enriched with stand age are negatively correlated with plant traits and soil nutrients, whereas the potential beneficial metabolites that depleted with stand age are positively correlated with plant traits and soil nutrients. Potential beneficial and harmful metabolites are negatively correlated with each other, whereas they exhibit positive correlations within their respective groups.

## 4 Discussion

This study identified triterpenoid saponins as the most abundant rhizosphere-enriched metabolites in alfalfa stands of different ages, followed by glycerolipids and fatty acyls. With increasing stand age, a number of triterpenoid saponins (potential autotoxins) were accumulated in both rhizosphere soils and plant roots, whereas glycerolipids, fatty acyls, and nucleotides (potential prebiotics) were all depleted. Our findings could facilitate the monitoring, prediction, and prevention of alfalfa decline under continuous cropping by harnessing functional metabolites as chemical markers and candidate prebiotics.

### 4.1 Biological decline of alfalfa is driven by autotoxins upregulated biosynthesis in roots and accumulation in rhizosphere soil

Triterpenoid saponins are a large group of triterpenoid glycosides formed by triterpenoid aglycones bound to various carbohydrate residues. These compounds are unique plant metabolites widely distributed in angiosperms (Tava et al., 2011). Triterpenoid saponins are known as allelopathic autotoxins in a variety of plants. For example, rhizosphere accumulation of ginsenosides jeopardizes plant health of continuously cropped *Panax notoginseng* (Burk.), a medicinal herb used in traditional Chinese medicine (Li et al., 2020). We found that the triterpenoid saponins accumulated in rhizosphere soils of the old alfalfa stand exhibited negative effects on plant growth, yield, and quality, as well as soil nitrogen nutrients. Medium to high concentrations of triterpenoid saponins represented by medicagenic acid and bayogenin inhibited alfalfa seed germination and seedling growth in Petri dishes. A similar inhibitory effect of medicagenic acid glycosides on seed germination has been observed in wheat (*Triticum aestivum* L.) (Oleszek and Jurzysta, 1987), cotton (*Gossypium hirsutum* L.) (Mishustin and Naumova, 1955, and barnyard grass (*Echinochloa crusgalli* L.) (Marchaim and Weker, 1974). Additionally, alfalfa seed germination was inhibited in the presence of root extracts at a low dilution. The root extract from old alfalfa plants showed a greater inhibitory effect than that of young plants, corresponding to higher accumulation of triterpenoid saponins in rhizosphere soils. The results provide preliminary evidence for the source of triterpenoid saponins in rhizosphere soils from alfalfa roots, and their accumulation with increasing stand age is one of the chemical mechanisms driving alfalfa decline.

The current understanding of triterpenoid saponin biosynthesis in alfalfa roots is still limited. Lei et al. (2019) characterized saponin profiles in various alfalfa ecotypes and observed root accumulation of bayogenin glycosides, hederagenin glycosides, soyaspogenin E glycosides, and medicagenic acid glycosides. We also found that a diverse set of triterpenoid saponins were accumulated in alfalfa roots of the old stand. Triterpenoid backbone biosynthesis is a major pathway for triterpenoid saponin biosynthesis, including the mevalonate pathway and methylerythritol 4-phosphate pathway (Yu et al., 2020). Mevalonate 5P involved in the mevalonate pathway showed higher accumulation in both the rhizosphere soil and alfalfa roots of the old stand compared to that in samples of the young stand. This metabolite was positively correlated with triterpenoid saponins in both the rhizosphere and root metabolomes. These results indicate that triterpenoid backbone biosynthesis in alfalfa roots is upregulated with age, which stimulates the root exudation of triterpenoid saponins and the accumulation of autotoxic metabolites in the rhizosphere. It has been reported that stress conditions enhance the root exudation of triterpenoid saponins from medicinal plants by stimulating the expression of genes associated with the terpenoid backbone biosynthesis pathway (Yang et al., 2022).

Galactose is an abundant and essential sugar used for the biosynthesis of many macromolecules in plants. Given the inhibitory effect of excess galactose and derivatives on plant growth, galactose metabolism is tightly and finely controlled (Wang et al., 2022b). We found that galactose metabolism was upregulated in alfalfa roots of the old stand. In particular, galactinol, melibiose, and sucrose were positively correlated with triterpenoid sapinins in alfalfa roots and rhizosphere soils, showing negative effects on most of the plant traits analyzed. These three metabolites were accumulated with increasing stand age, potentially raising the risk of plant diseases. For example, melibiose is closely related to the early development of strawberry gray mold (Hu et al., 2019). Galactinol, melibiose, and sucrose are specifically used as nutrients by plant pathogenic bacteria, such as *Agrobacterium fabrum* (Meyer et al., 2018). Furthermore, uridine is a nucleotide in close association with the composition of RNA, and it plays a vital role in many physio-biochemical processes of organisms (Sun et al., 2021). Uridine accumulation in rhizosphere soils was enhanced with increasing stand age of alfalfa, and this metabolite showed negative effects on alfalfa yield and quality in terms of crude fiber and neutral detergent fiber contents. Given its positive correlation with triterpenoid saponins, uridine is likely an autotoxin contributing to alfalfa decline, but its source and exact mechanism still need to be further investigated.

### 4.2 Biological decline of alfalfa is attributed to probiotics downregulated biosynthesis in roots and decrease in rhizosphere soil

Glycerolipids, including glycerophospholipids and glyceroglycolipids, are major cell membrane components and signaling molecules. Glycerolipids take part in a wide range of physio-biochemical processes, contributing to plant growth and development (Ke et al., 2023). Specifically, glycerophospholipids are oversynthesized in plants under stress conditions to maintain cell membrane stability and function and glyceroglycolipids closely relate to plant photosynthesis (Li et al., 2023). The glycerolipids detected in rhizosphere soils and alfalfa roots were negatively correlated with triterpenoid saponins, showing positive effects on most of the plant traits analyzed in the present study. This indicates that glycerolipids may serve as potential probiotics contributing to alfalfa growth. With respect to glycerophospholipid metabolism, 2-lysolecithin and glycerophosphocholine were depleted in alfalfa roots with increasing stand age, consistent with the pattern of most glycerolipids in rhizosphere soils. This provides evidence for age-related downregulation of probiotic biosynthesis in alfalfa roots of the old stand, which is one of the possible reasons for alfalfa decline, although the underlying mechanism is still elusive. Ma et al. (2017) found that the glyceroglycolipid content in rice leaves decreased under stress conditions, resulting in lower chlorophyll content. This allowed us to posit that substantial accumulation of triterpenoid saponins in the rhizosphere of old alfalfa plants jeopardizes root health, inhibiting glyceroglycolipid biosynthesis. The consequent decrease in chlorophyll content weakens plant photosynthesis, leading to inferior growth of alfalfa.

Fatty acyls play a crucial role in the formation and development of rhizomes in legume crops. These compounds serve as carbon substrates and perform signaling functions in the nitrogen cycle, regulating soil–root interactions in soybean (Gao et al., 2022). The fatty acyls identified in rhizosphere soils and alfalfa roots showed positive effects on the majority of plant traits and soil nitrogen nutrients. Taking into account their negative correlations with triterpenoid saponins, these fatty acyls are potential prebiotics contributing to alfalfa growth. Furthermore, α-linolenic acid metabolism is essential for plant stress resistance (Fernandes and Ghag, 2022; Fu et al., 2023). Both traumatic acid and colnelenic acid related to α-linolenic acid metabolism were depleted in rhizosphere soils and alfalfa roots of the old stand. This means age-related downregulation of α-linolenic acid metabolism in alfalfa roots, which could reduce the biosynthesis of prebiotic metabolites such as fatty acyls, weakening positive root–soil interactions.

Nucleotides participate in intracellular signaling, regulate plant growth and reproduction, and trigger plant immune responses (Claus and Marco, 2024). Among the rhizosphere metabolites involved in nucleotide metabolism, both adenosine and deoxyguanosine positively affected a variety of alfalfa plant traits and negatively correlated with triterpenoid saponins, galactoses, and uridine. Adenosine contributes to plant growth through its participation in cellular energy metabolism (Peter et al., 2010). Adenosine is also involved in apoptosis and sphingolipid signaling, which are important pathways for regulation of cell survival and resistance (Zhu et al., 2024; Ali et al., 2018). Deoxyguanosine has been reported as a novel immune signaling molecule that efficiently mediates plant immune resistance (Lu et al., 2023). Therefore, adenosine and deoxyguanosine represent prebiotic metabolites that confer benefits to alfalfa growth. These metabolites were depleted in both rhizosphere soils and alfalfa roots of the old stand, suggesting age-related downregulation of nucleotide metabolism in the roots, which could hinder molecular signaling and impair plant development. The results underscore the weakening of nucleotide metabolism in alfalfa roots with increasing stand age, accompanied by downregulated biosynthesis of potential prebiotics (e.g., adenosine), which causes the deterioration of soil chemical environment in the rhizosphere.

## 5 Conclusions

**Figure 8:**
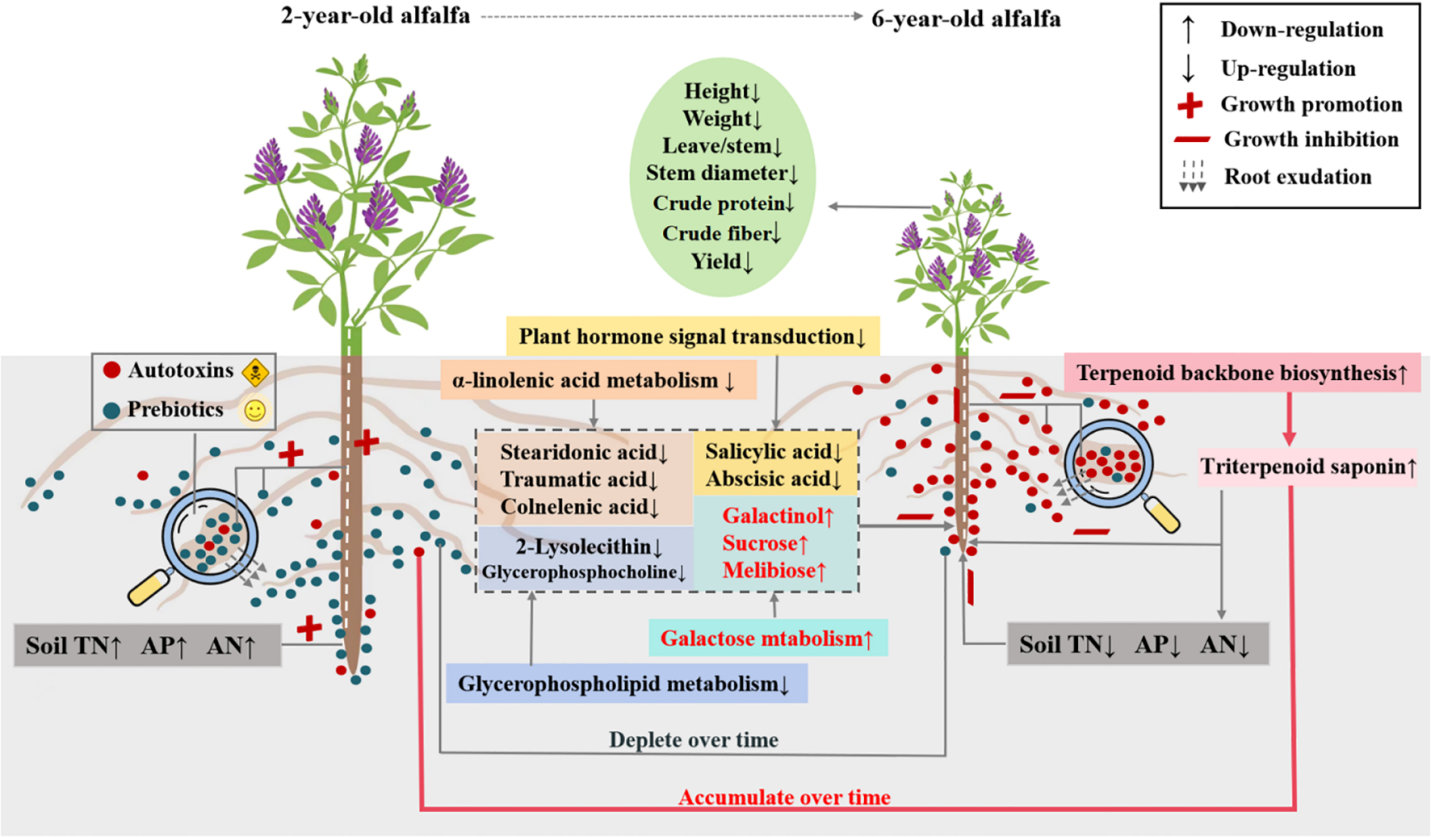
Schematic diagram of soil-plant metabolic crosstalk underlying alfalfa decline. TN, total nitrogen; AP, available phosphorus; AN, alkali-hydrolyzable nitrogen.

This study showed age-induced changes in rhizosphere and root metabolomes, emphasizing the role of functional metabolites in alfalfa decline through plant-soil crosstalk. Stand age affected the pathways of terpenoid backbone biosynthesis and plant hormone signal transduction, as well as metabolism of galactose, glycerophospholipid, α-linolenic acid, and caffeine in alfalfa roots. The biosynthesis of autotoxins (e.g., triterpenoid saponins) with inhibitory effects on seed germination was upregulated in alfalfa roots of old plants, accompanied by downregulated biosynthesis of prebiotics (e.g., glycerolipids, fatty acyls, and nucleotides) related to stress resistance, growth promotion, and signal transduction. Root exudation from old plants led to considerable accumulation of autotoxins in rhizosphere soils, with concomitant depletion of prebiotics. As a consequence, the soil chemical environment in the rhizosphere was deteriorated, leading to alfalfa decline (**Figure 8**). This study uncovers the mechanism of alfalfa decline mediated by plant and soil metabolic reprogramming, which could foster the development of chemical markers and prebiotics for detection and prevention purposes.

## Data Availability

The original contributions presented in the study are included in the article/**Supplementary Material**. Further inquiries can be directed to the corresponding author.
