# Supplementary material for: Metabolic crosstalk between roots and rhizosphere drives alfalfa decline under continuous cropping
Source: Front Plant Sci. 2024 Dec 12;15:1496691. doi: 10.3389/fpls.2024.1496691 (PMC11670254; doi:10.3389/fpls.2024.1496691)
Supplement: Supplementary file 1 [file Table1.docx]

Supplementary Material

**Supplementary TABLE S1** Relative abundance of potential prebiotic and autotoxins in rhizosphere soils of different stand ages（×10^3^）

| **No.** | **Metabolite** | **Class** | **6R** | **2R** | **Fold Change** |
| --- | --- | --- | --- | --- | --- |
| 1 | PIP(16:0/18:0) | Glycerolipids | 34 | 57 | 0.6^＊＊^ |
| 2 | PE(16:1(9Z)/22:6(4Z,7Z,10Z,13Z,16Z,19Z)) |  | 1 | 32 | 0.02^＊^ |
| 3 | PC(14:1(9Z)/P-18:1(11Z)) |  | 4 | 8 | 0.4^＊^ |
| 4 | Sulfatide |  | 8 | 13 | 0.6^＊^ |
| 5 | Glycerol 1-hexadecanoate |  | 612 | 4300 | 0.1^＊＊^ |
| 6 | 1-Monopalmitin |  | 20 | 124 | 0.2^＊＊^ |
| 7 | DG(13:0/12:0/0:0) |  | 8 | 28 | 0.3^＊＊^ |
| 8 | DG(14:1(9Z)/14:1(9Z)/0:0) |  | 3 | 6 | 0.5^＊^ |
| 9 | MG(0:0/14:0/0:0) |  | 104 | 191 | 0.5^＊＊^ |
| 10 | Traumatic acid | Fatty Acyls | 10 | 60 | 0.2^＊＊^ |
| 11 | Colnelenic acid |  | 18 | 77 | 0.2^＊^ |
| 12 | 12-Hydroxy-12-octadecanoylcarnitine |  | 35 | 104 | 0.3^＊＊^ |
| 13 | Citronellyl acetate |  | 1126 | 3234 | 0.3^＊＊^ |
| 14 | Stearic acid |  | 170 | 340 | 0.5^＊^ |
| 15 | O-Arachidonoyl Ethanolamine |  | 14 | 26 | 0.6^＊^ |
| 16 | Dibutyl adipate |  | 45 | 79 | 0.6^＊^ |
| 17 | xi-8-Hydroxyhexadecanedioic acid |  | 20 | 32 | 0.6^＊^ |
| 18 | Tween 20 |  | 35 | 53 | 0.7^＊＊^ |
| 19 | Deoxyguanosine | Purine nucleosides | 55 | 80 | 0.3^＊＊^ |
| 20 | Adenosine |  | 15 | 18 | 0.5^＊^ |
| 21 | Uridine |  | 204 | 41 | 5^＊^ |
| 22 | Mevalonate-5P | Phosphate  esters | 96 | 50 | 1.9^＊^ |
| 23 | Galactinol | Galactose | 2250 | 646 | 3.5^＊^ |
| 24 | Sphingosine | Amines | 23 | 42 | 0.6^＊＊^ |

* P < 0.05 and ** P < 0.01 (Student's t test; n = 6).
