# Supplementary material for: Metabolic crosstalk between roots and rhizosphere drives alfalfa decline under continuous cropping
Source: Front Plant Sci. 2024 Dec 12;15:1496691. doi: 10.3389/fpls.2024.1496691 (PMC11670254; doi:10.3389/fpls.2024.1496691)
Supplement: Supplementary file 2 [file Table2.docx]

Supplementary Material

**Supplementary TABLE S2** Relative abundance of potential prebiotic and autotoxins in alfalfa roots of different stand ages（×10^3^）

| **No.** | **Metabolite** | **Class** | **Old**  **stand** | **Young**  **stand** | **Fold**  **Change** |
| --- | --- | --- | --- | --- | --- |
|  |  |  |  |  |  |
| 1 | ※PIP(20:1(11Z)/18:2(9Z,12Z)) | Glycerolipids | 3 | 61 | 0.05^＊＊^ |
| 2 | ※PIP(18:1(11Z)/18:3(6Z,9Z,12Z)) |  | 4 | 27 | 0.1^＊＊^ |
| 3 | ※LysoPC(20:2(11Z,14Z) |  | 7 | 28 | 0.3^＊＊^ |
| 4 | ※LysoPE(18:1(11Z)/0:0) |  | 9 | 25 | 0.4^＊＊^ |
| 5 | PIP2(22:4(7Z,10Z,13Z,16Z)/16:0) |  | 2 | 10 | 0.2^＊^ |
| 6 | PA(18:3(6Z,9Z,12Z)/0:0) |  | 16 | 38 | 0.4^＊^ |
| 7 | LPE(15:0) |  | 64 | 137 | 0.5^＊＊^ |
| 8 | PC(20:2(11Z,14Z)/18:3(9Z,12Z,15Z)) |  | 107 | 230 | 0.5^＊^ |
| 9 | PA(20:5(5Z,8Z,11Z,14Z,17Z)/18:4(6Z,9Z,12Z,15Z) |  | 7 | 15 | 0.5^＊^ |
| 10 | PC(18:2(9Z,12Z)/15:0) |  | 56 | 109 | 0.5^＊^ |
| 11 | PC(30:0) |  | 38 | 66 | 0.6^＊^ |
| 12 | 2-Lysolecithin |  | 8 | 26 | 0.3^＊＊^ |
| 13 | Glycerophosphocholine |  | 42 | 129 | 0.3^＊^ |
| 14 | Isocaproic acid | Fatty Acyls | 8 | 36 | 0.2^＊＊^ |
| 15 | Heneicosanoic acid |  | 40 | 96 | 0.4^＊^ |
| 16 | 2,3-Dinor-11b-PGF2a |  | 39 | 84 | 0.5^＊^ |
| 17 | 9-HODE |  | 223 | 474 | 0.5^＊^ |
| 18 | Stearidonic acid |  | 10 | 20 | 0.5^＊^ |
| 19 | ※MG(18:4(6Z,9Z,12Z,15Z)/0:0/0:0) |  | 147 | 259 | 0.6^＊^ |
| 20 | Azelaic acid |  | 109 | 186 | 0.6^＊＊^ |
| 21 | ※Traumatic acid |  | 111 | 203 | 0.5^＊^ |
| 22 | ※Colnelenic acid |  | 27 | 88 | 0.3^＊＊^ |
| 23 | ※Adenosine | Purine nucleosides | 3899 | 4587 | 0.9 |
| 24 | ※Deoxyguanosine |  | 27 | 41 | 0.7 |
| 25 | ※Uridine |  | 70 | 207 | 0.3^＊^ |
| 26 | Melibiose | Galactose | 7873 | 4763 | 1.7^＊^ |
| 27 | ※Galactinol |  | 7873 | 4763 | 1.7^＊^ |
| 28 | Sucrose |  | 7873 | 4763 | 1.7^＊^ |
| 29 | Salicylic acid | Plant hormone | 77 | 241 | 0.3^＊＊^ |
| 30 | Abscisic acid |  | 8 | 16 | 0.5^＊^ |
| 31 | Mevalonate-5P | Phosphate esters | 114 | 74 | 1.5^＊^ |

* P < 0.05 and ** P < 0.01 (Student's t test; n = 6). ※indicates the metabolites also detected in rhizosphere soils.
